# Study of Relaxations in Epoxy/Rubber Composites by Thermally Stimulated Depolarization Current and Dielectric Spectroscopy

**DOI:** 10.3389/fchem.2022.874685

**Published:** 2022-03-17

**Authors:** Chuang Wang, Gang Zhou, Weiyu Zhu, Chi Chen, Yuwei Fu, Zaiqin Zhang, Hui Li

**Affiliations:** School of Electrical Engineering, Xi’an University of Technology, Xi’an, China

**Keywords:** epoxy resin, interfacial polarization, trap depth, HTBN, relaxation characteristics

## Abstract

Liquid rubber toughened epoxy resins are widely used in electrical equipment and electronic packaging. Previous studies have only investigated the relaxation process of epoxy resins through dielectric spectroscopy. The trap characteristics of the relaxation process by thermally stimulated depolarization current (TSDC) analysis are less studied. In this work, TSDC and broadband dielectric spectroscopy techniques were used to complementarily characterize the dielectric relaxation process of hydroxyl-terminated liquid nitrile-butadiene rubber (HTBN) toughened epoxy resin polymers. The experimental results show that HTBN introduces two new relaxation processes in the epoxy matrix, which are attributed to the α polarization of the rubber molecule and the interfacial polarization based on the correlation between the TSDC and the dielectric spectroscopy data, respectively. The trap parameters of each TSDC current peak were obtained using the multi-peak fitting method. The addition of rubber increases the trap density in epoxy composites significantly, especially for traps with energy levels in the range of 0.5–0.9 eV. The trap energy level of the DC conductivity process increases with increasing rubber concentration. The above results provide analytical ideas for rubber-toughened epoxy resins’ polarization and trap characteristics and theoretical guidance for formulation improvement.

## Introduction

Epoxy resins are widely used in power equipment and electronic devices because of their excellent adhesion, corrosion resistance, electrical insulation and ease of processing and forming (Rimdusit and Ishida, 2000; Cao et al., 2015; Jin et al., 2015). However, as a thermosetting material, epoxy resin has a highly cross-linked internal structure, which leads to a high brittleness and is susceptible to fracture and insulation damage in high-stress situations, limiting its application scope (Soares et al., 2011). In addition, good dielectric properties enable long-term safe and stable operation of power electronics. Therefore, epoxy resins need to be toughened in many applications to improve their mechanical and dielectric properties (Wang et al., 2021a; Wang et al., 2021b; Wang et al., 2022a; Wang et al., 2022b).

Studies have shown that liquid rubber doped epoxy resin can effectively improve the mechanical and dielectric properties. The commonly used liquid rubbers are carboxyl-terminated liquid nitrile-butadiene rubber (CTBN), HTBN, hydroxyl-terminated liquid polybutadiene rubber (HTPB) and carboxyl-terminated liquid polybutadiene rubber (CTPB) (Tripathi and Srivastava, 2007; Minfeng et al., 2008; Zhou and Cai, 2012; Dong et al., 2016). Dong and his co-workers (Dong et al., 2016) modified epoxy resin by adding CTPB. They found that CTPB-modified EP has higher resistivity and breakdown strength, lower dielectric constant and loss compared with pure epoxy resin (EP). Zhou and Zuo (2013) investigated the effect of adding HTPB on the mechanical and electrical properties of epoxy resins. It was found that the addition of 10% mass fraction of HTPB resulted in the best tensile and flexural properties. The addition of HTPB reduced the thermal stability of the material, but significantly increased the bulk and surface resistivity. Wang et al. (2016) toughened the epoxy resin using HTBN as filler. The addition of HTBN resulted in little change in dielectric strength, a slight decrease in volume resistivity and glass transition temperature, and an increase in relative dielectric constant. In addition, the interfacial polarization between rubber particles and epoxy resin makes a peak on the dielectric loss curve. They also investigated the dielectric properties of epoxy resins toughened by liquid rubber with different polarities. It was found that reducing the polarity of liquid rubber filler could reduce the relative dielectric constant and dielectric loss of epoxy resin/liquid rubber composites by broadband dielectric spectrum analysis (Wang et al., 2020a). However, the above studies only discussed the relaxation process using dielectric spectroscopy, and the analysis of the trap properties was rarely addressed.

Since epoxy resin materials are primarily applied in complex physical fields such as electricity, heat and force, which are prone to aging causing more physical and chemical defects. The dielectric spectroscopy technique cannot reflect the trap properties of the material, and scholars have investigated the connection between polarization and trap distribution using the TSDC method (Smaoui et al., 2009; Katayama et al., 2013; Krentz et al., 2014; Ning et al., 2015a). J. Katayama and his colleagues compared the effects of different nanofillers on the TSDC of epoxy resins. The results show that the low temperature peak in TSDC is clearly caused by dipole depolarization due to the glass transition, while the high temperature peak is caused by the release of space charge. Various nanofillers affect the two peaks to different degrees, which is attributed to the inhibition of molecular motion by the nanofillers (Katayama et al., 2013). Broadband dielectric spectroscopy and TSDC technique are two complementary techniques often used to obtain the complete knowledge of relaxation and conduction mechanisms, especially when multiple relaxation processes are superimposed on each other. The combination of these techniques allows a more comprehensive study of the relaxation processes. H Smaoui et al. (Smaoui et al., 2009) investigated the dielectric relaxation and molecular mobility of ZnO nanoparticle-filled epoxy nanocomposites using broadband dielectric relaxation spectroscopy and TSDC techniques. It was shown that the non-homogeneity introduced by the nanofiller particles increased the space charge density of the epoxy nanocomposites. F Namouchi et al. adopted (Namouchi et al., 2015) to characterize the dielectric relaxation process of poly (methyl methacrylate) (PMMA) polymers by TSDC and broadband dielectric relaxation spectroscopy techniques for dipole β and α relaxation and interfacial ρ relaxation. The results showed that the energy levels of β, α and ρ peaks were obtained by curve fitting as 0.65–0.88 eV, 1.28–2.05 eV and 2.1–2.58 eV, respectively. However, most of the above studies used nano-fillers, and the formation mechanism of the interface between nano-fillers and the epoxy matrix is different from that of rubber. It is necessary to study the dielectric spectroscopy jointly and TSDC of liquid rubber toughened epoxy resins to understand the mechanism of each relaxation process and provide a theoretical basis for the design of formulation systems.

In this paper, TSDC and broadband dielectric spectroscopy techniques were used to complementarily characterize the dielectric relaxation process of HTBN toughened epoxy resin polymers. The relaxation process corresponding to each current peak was analyzed by splitting the TSDC current peak and verified using the dielectric spectrum data. The trap parameters of each current peak were calculated, and the effect of the introduction of HTBN on the trap characteristics of the relaxation process of epoxy resin was studied. The study results can provide analytical ideas for the polarization and trap characteristics of rubber-toughened epoxy resins and theoretical guidance for formulation improvement.

## Materials and Methods

### Preparation Sample

The epoxy resin used in this study is diglycidyl ether of bisphenol A (DGEBA) with the epoxy value of 3.9 mmol/g, which was produced by Xingchen Synthetic Materials Co., Ltd., Nantong, China. The curing agent was polyoxide propylene diamine, a room temperature amine curing agent, purchased from purchased from Zhongsi Industrial Co. Ltd., Shanghai, China. And the average molecular weight (Mn) adopted in our research is 400. The liquid rubber was selected as terminal hydroxyl liquid nitrile butadiene rubber with a hydroxyl content of 0.60 mmol/g. All reagents were not specially treated before use.

Samples of different rubber contents were prepared according to the following steps. First, HTBN was added to the epoxy resin and the mixture was homogeneously mixed, followed by low-speed stirring in an oil bath at 150°C to make it homogeneously mixed. After 1 h, the blends were removed and cooled naturally, and then the curing agent was added, stirred homogeneously, and then vacuum degassed. The prepolymer was injected into the mould under vacuum environment and then left for 2 h at room temperature for initial curing, followed by placing the mould in an oven and curing under the conditions of 40°C/2 h, 60°C/2 h, 80°C/3 h and 110°C/4 h. After curing, the temperature was cooled down for the polymer gradient and then removed from the oven.

### Performance Measurement

The dielectric spectrum measurements were performed on the specimens using a Concept80 dielectric spectrum tester from Novocontrol, Germany. The size of the sample is 40 mm in diameter and 1 mm in thickness. By the ion sputtering, one side of the sample is coated with gold all, and the other side is coated with a circle of 30 mm. The measurement frequency range was 0.1 Hz–10^6^ Hz, and the temperature range was -60–200°C.

**FIGURE 1:**
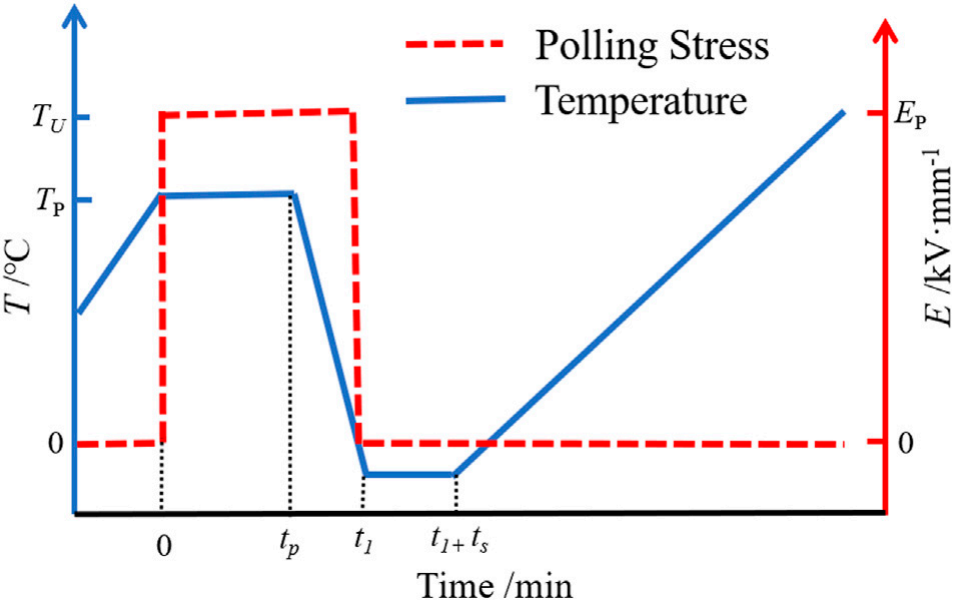
Schematic of TSDC program on epoxy/rubber composite.

**FIGURE 2:**
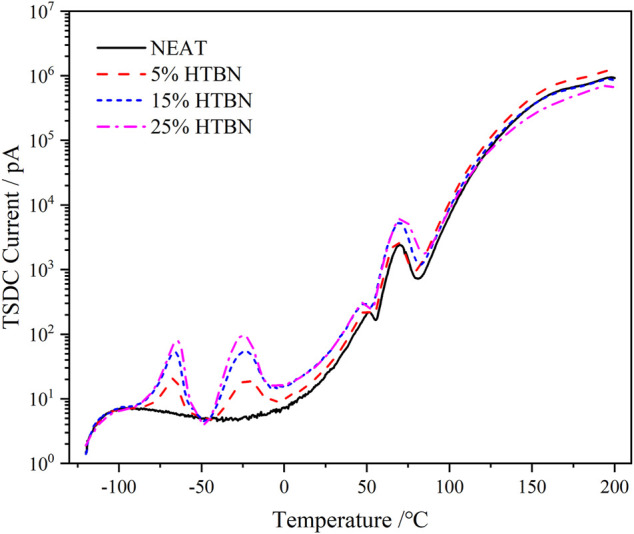
TSDC spectra of samples with different rubber contents.

TSDC measurement has been performed to determine the trap depth and density in the epoxy/rubber composites. Both sides of vacuum dried samples were sputtered with golden electrodes with a diameter of 30 mm. The tests have been carried out with a Novocontrol Quatro Cryosystem. TSDC experimental scheme is shown in Figure 1. Before the test, the specimens were sputtered with gold electrodes on both sides and dried under vacuum (100°C, 50 Pa) for 13 h to remove as much water as possible from the interior of the specimens. As shown in Figure 2, the test process is: when the temperature rises to the polarization temperature *T*_p_ (160°C), then maintain the temperature constant. *E*_p_ (0.3 kV/mm) DC polarization field strength is applied to both ends of the specimen for a duration of *t*_p_ (40 min). Then the specimen temperature is rapidly reduced to −120°C, when the applied voltage is withdrawn and the specimen is short-circuited at both ends for *t*_p_ (5 min). Finally, the temperature was increased linearly at 3°C/min, while the current in the external circuit was measured using a Keithley 6517B electrostatic meter, which is the depolarization current.

### Principles of Thermally Stimulated Depolarization Current Peak Trap Parameter Calculation

The calculation of the thermal stimulation current is complex, and the trap parameters are closely related to the thermal motion of the molecules. The following kinetic equation can describe the thermal stimulation process.

$$I\left(t\right)=-\frac{dn}{dt}={\left(\frac{n}{n_{0}}\right)}^{b}sn_{0}\exp\left(-\frac{E}{kT}\right)$$ (1)

where *n* is the carrier concentration in the trap, *s* is the frequency factor, *n*_0_ means the initial concentration of carriers in the trap, *E* is the activation energy, *k* is the Boltzmann constant, *T* is the absolute temperature, and *b* is the kinetic level.

The solution of Equation 1 is given by:

$$I\left(T\right)={\text{n}}_{0}\text{s}\exp\left(-\frac{E}{kT}\right)\exp(-\frac{s}{\beta }\int_{T_{0}}^{T}e^{-\frac{E}{kT}}dT^{\prime }\left(b=1\right)$$ (2)

$$1+\frac{\left(b-1\right)s}{\beta }\int_{T_{0}}^{T_{m}}e^{-\frac{E}{kT}}dT^{\prime }=\frac{bskT_{\text{m}}^{2}}{\beta E}e^{-\frac{E}{kT}}\left(b\neq 1\right)$$ (3)

If a linear temperature rise is used during the experiment, the following equation can be derived.

$$\frac{\beta E}{kT_{m}^{2}}=se^{-\frac{E}{kT_{m}}}\left(b=1\right)$$ (4)

$$1+\frac{\left(b-1\right)s}{\beta }\int_{T_{0}}^{T_{m}}e^{-\frac{E}{kT}}dT^{\prime }=\frac{bskT_{\text{m}}^{2}}{\beta E}e^{-\frac{E}{kT}}\left(b\neq 1\right)$$ (5)

where *β* is the linear rate of temperature increase, and *T*_m_ is the temperature corresponding to the peak current. Taking the logarithm of Equation 1 and doing any three data points on the TSDC curve to make the difference, we can obtain the following equation.

$$E=\frac{k\left[\ln\left(\frac{I_{3}}{I_{1}}\right)\ln\left(\frac{n_{2}}{n_{1}}\right)-\ln\left(\frac{I_{2}}{I_{1}}\right)\ln\left(\frac{n_{3}}{n_{1}}\right)\right]}{\left(\frac{1}{T_{2}}-\frac{1}{T_{1}}\right)\ln\left(\frac{n_{3}}{n_{1}}\right)-\left(\frac{1}{T_{3}}-\frac{1}{T_{1}}\right)\ln\left(\frac{n_{2}}{n_{1}}\right)}$$ (6)

$$b=\frac{\ln\left(\frac{I_{2}}{I_{1}}\right)+\frac{E}{k}\left(\frac{1}{T_{2}}-\frac{1}{T_{1}}\right)}{\ln\left(\frac{n_{2}}{n_{1}}\right)}$$ (7)

$$n=\frac{1}{\beta }\int_{T}^{T_{\infty }}IdT^{\prime }$$ (8)

The integrals for Equations 2 and 8 are calculated using Gaussian quadrature and segmented cubic spline interpolation, respectively (Lei et al., 1992; Fan et al., 1999). The characteristic parameters of the TSDC separation peak can be obtained according to the above equation.

### Thermally Stimulated Depolarization Current Spectrum Analysis of Hydroxy Liquid Nitrile Rubber Modified Epoxy Resin

Figure 2 shows the TSDC spectra of samples with different rubber contents. The curves contain the superposition of several current peaks in the whole temperature range, where the current depolarization peaks increase with the rubber content between −100 and 120°C. Above 120°C, the current peaks increase and decrease with the rubber content. In order to obtain the characteristic parameters of each current peak, the TSDC needs to be divided into peaks.

**FIGURE 3:**
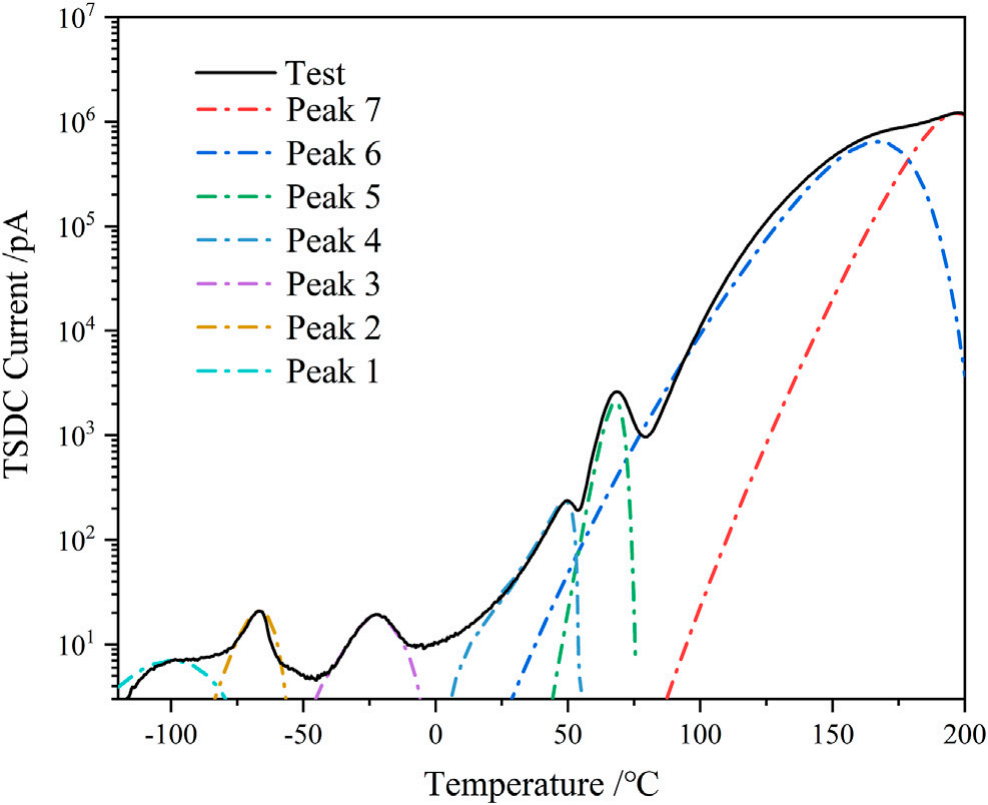
Multi-peak fitting of TSDC spectrum for 5% HTBN samples.

According to the derivation in Section 3, as shown in Figure 3, the TSDC increases with temperature, and we start with a multi-peak fitting to the current peak at high temperatures. The characteristic parameters of peak seven are calculated by Equations 6 and 7 by selecting the appropriate calculation points. The isolated peak seven curve is then subtracted from the composite TSDC curve, so that the falling edge curve of peak 6 can be obtained, and the parameters of peak 6 can be found. By analogy, all the individual peaks are calculated, and the 5% HTBN depolarization current curve, for example, can be divided into seven individual peaks.

### Effect of Hydroxy Liquid Nitrile Rubber Introduction on Low-Temperature Dielectric Relaxation of Epoxy Resins

**FIGURE 4:**
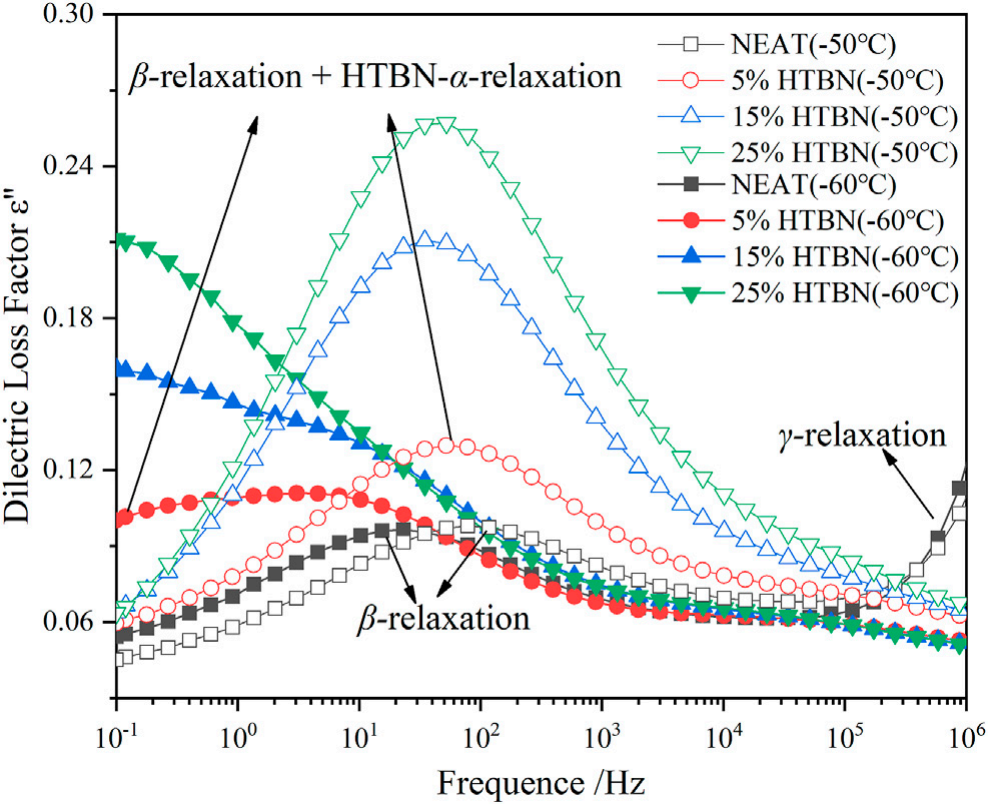
Variation of dielectric loss factor versus frequency for samples with different rubber content at low temperature.

Figure 4 shows the imaginary curves of dielectric constants for different rubber content samples in the low temperature range, the epoxy resin shows a clear secondary transition, which is the β-relaxation process. HTBN addition shows a relaxation peak of greater intensity at −60°C and −50°C, while the positions of the 2 relaxation peaks are not consistent with the β-relaxation of the epoxy. At −60°C, the addition of HTBN made the width of the relaxation peak at the low-frequency position larger. Combined with the analysis of several curves, the relaxation peak in the low temperature region after the addition of HTBN should be two relaxation processes superimposed on each other. Based on the location and temperature of this relaxation peak, it can be assumed that the chain segment motion of HTBN molecules obscures the relaxation process of the secondary transition process of the epoxy itself. Compared with pure epoxy, the dielectric loss factor increases by a factor of 1–2 after the addition of HTBN, which indicates that the intensity of α relaxation of HTBN molecules is greater than the secondary transition of epoxy molecules, as reported in other literature (Wang et al., 2020b).

As shown in the TSDC curves in Figure 2, two additional peaks can be clearly found in the low-temperature section for the samples containing rubber filler compared to the pure epoxy resin, which is peak 2 in the temperature range of −100 ∼ −50°C and peak 3 in the range of −50–0°C. Since these two peaks do not appear in the pure epoxy resin samples, it indicates that the addition of HTBN causes the polarization process. The relative permittivity spectra of this sample in the low-temperature section were reported in the literature (Chen et al., 2020), and the relative permittivity of each sample was close at −60°C. This indicates that the difference between the dielectric properties of the epoxy matrix and the rubber filler in the low-temperature section is slight, and the interfacial polarization is low. Hence, −100 ∼ −50°C is the current peak caused by the α polarization of the rubber molecule.

Moreover, the temperature below −100°C, all samples have the same change trend. There is a broader current peak, which is judged to be caused by β relaxation of epoxy resin according to the peak’s size and position, consistent with the literature (Ning et al., 2015a). The above judgment of the type of polarization in the low-temperature section is consistent with the results of dielectric spectrum analysis.

Since the glass transition peak of HTBN is close to the epoxy secondary transition peak in the dielectric spectrum, meanwhile the epoxy secondary transition peak is gradually masked as the rubber content increases. To further analyze the trap characteristics of dielectric relaxation at low temperatures, the trap energy levels of β relaxation and α relaxation of rubber molecules were calculated for different samples according to the relevant results in Figure 3. As can be seen from the table, the trap energy level of β relaxation is unchanged after HTBN. Furthermore, the literature (Ning et al., 2015b) reported that the trap energy level of epoxy secondary transition decreases with insulating paper content. Because the difference in the chemical composition of rubber and insulating paper makes the two have different degrees of influence on the curing cross-linking reaction of epoxy resin, and the cross-linking process affects the strength of secondary transition. While the analysis of the dielectric spectrum in literature (Wang et al., 2020b) mentions that the addition of HTBN increases the hydroxyl group in the epoxy matrix, which enhances the β process, this trend is not reflected in the magnitude of the depolarization current peak in this paper. This may be because the test polarization temperature is high and the polarization process at low temperatures is not obvious. The α relaxation trap energy level of rubber molecules increases with increasing rubber content due to the incorporation of rubber that causes traps including physical defects, chemical defects, and introduced impurities (Syed et al., 2016). For example, the large number of broken chains branched chains, and some impurities in the rubber molecule make the presence of more traps of varying depths inside.

### Effect of Hydroxy Liquid Nitrile Rubber Introduction on the Interfacial Polarization of Epoxy Resin

As the curing cross-linking reaction proceeds, the molecular weight of the epoxy resin increases, making the compatibility between the two poor. The unstable phase separation mechanism separates the rubber phase and the epoxy resin phase and gradually precipitate, forming an intercalated phase dispersed in the cured epoxy resin (Thomas et al., 2007). At the same time, impurity ions in the system are precipitated at the phase boundary site. Since the mobility of carriers in the rubber phase is greater than their mobility in the cured epoxy resin, the motion process of carriers under external electric field can be depicted by MWS polarization process. According to the Maxwell-Wagner effect of dielectric, when two dielectrics are in contact, if the charge is subjected to directional motion by the external electric field, the difference in the dielectric constants of the materials will result in different charge movement at their interfaces. The charge near the interface will start to accumulate, which will produce relaxation-type polarization due to consuming energy and time (Hammami et al., 2007).

**FIGURE 5:**
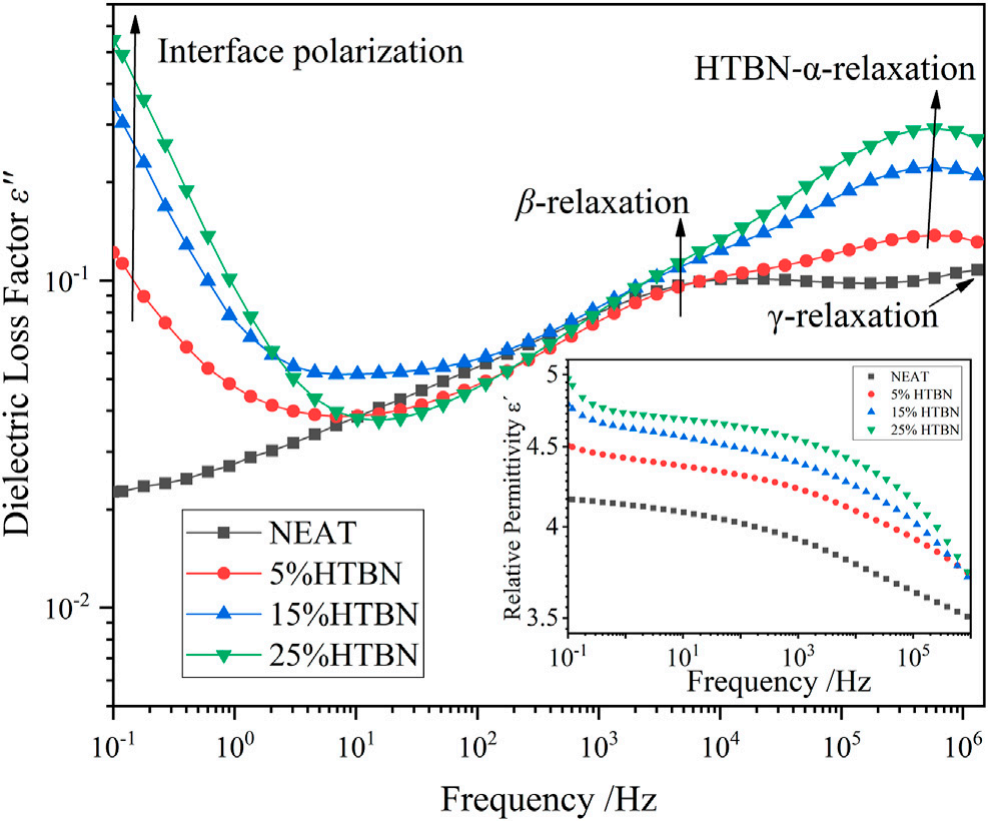
Dielectric properties of different rubber contents at −10°C.

**FIGURE 6:**
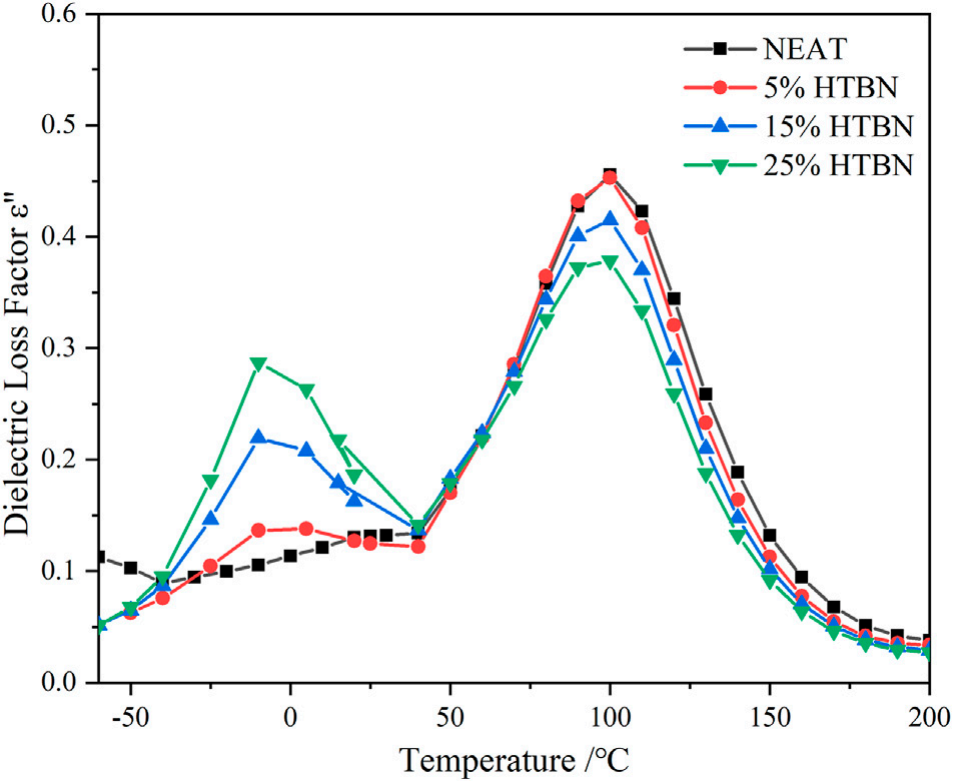
Temperature dependence of the imaginary part of the complex dielectric constant of different rubber contents at high frequency.

According to the dielectric spectrum analysis in Figure 5, the loss peak caused by interfacial polarization already appears at −10°C in the low frequency band. Figure 6 shows the change of dielectric loss with temperature for different rubber contents at high frequencies. At −20°C, the samples with rubber filler differ from pure epoxy by an obvious dielectric loss peak, and the higher the rubber content, the larger the loss peak. The loss peak was judged to be caused by interfacial polarization according to its temperature and frequency (Chen et al., 2020). In summary, combined with the analysis in Section 4.1, in the TSDC spectrum, −50–0°C is the interfacial polarization caused by the dielectric properties between rubber filler and epoxy matrix.

At present, the influence of rubber molecules on the dielectric properties of epoxy resins is mainly studied in academia by introducing interfacial properties (Zhou and Cai, 2012; Zhou and Zuo, 2013; Wang et al., 2016; Wang et al., 2020a). It has been suggested that the long flexible chains of rubber can extend to the particles’ outer layer and enter into the cross-linked network in the reaction with the epoxy resin. This process is similar to the formation of complex interfacial structures between inorganic nanoparticles and the polymer matrix due to their intense surface activity, leading to the use of the interaction zone model to explain the interfacial properties (Tanaka et al., 2005).

**FIGURE 7:**
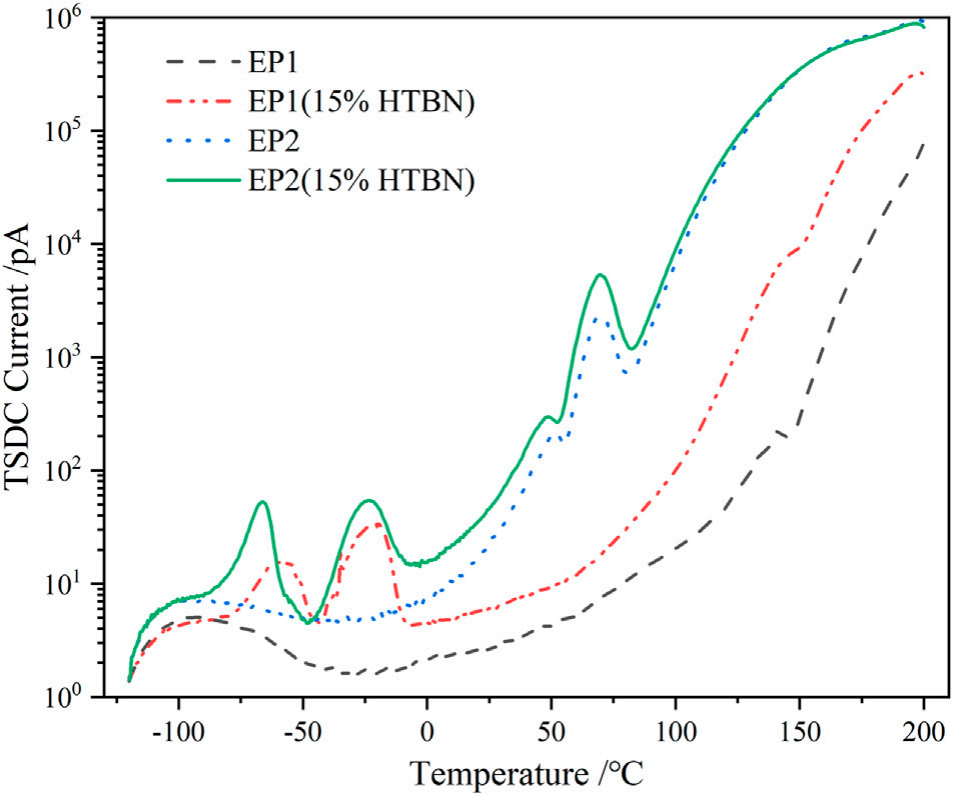
TSDC spectra under different formulations.

Rubber molecules entering the epoxy resin matrix become entangled and chained into spherical (or sphere-like) particles on the nanometer (or micron) scale, and the number of particles increases with increasing rubber content (Wang et al., 2016). The particles are bound internally by intermolecular interaction forces, the magnitude of which determines the size of the rubber particles and is also influenced by external factors such as temperature during curing. There are differences in the size of the rubber formed by phase separation of different epoxy curing formulations (Wang et al., 2020a). The interfacial properties formed by the HTBN and epoxy matrix used in this paper are mainly related to the degree of bonding between the rubber particles and the epoxy matrix. Of course, the interfacial polarization of the epoxy matrix and rubber molecules is also related to other factors, including curing conditions, and rubber molecule type. The interfacial polarization activation energy of rubber with four different polarities was analyzed in the literature (Wang et al., 2020a). As the polarity of liquid rubber increases, the overall activation energy increases, which indicates that the polarity of rubber molecules affects the ease of interfacial polarization. In addition, interfacial polarization is also influenced by the temperature dependence of the rubber phase conductivity. Two epoxy formulations with different curing agents were used to study the degree of interfacial bonding precisely and to study the effect of HTBN on the trap properties of the epoxy resin. EP1 was cured with a methyl-hexahydro phthalic anhydride curing agent, and EP2 adapts polyoxypropylene diamine curing agent. Since the main difference between the two epoxy formulations is the curing temperature, EP1 is a high temperature curing formulation with a glass transition temperature of about 140°C, and EP2 is a low temperature curing formulation with a glass transition temperature of about 70°C. As shown in Figure 7, the trend of depolarization current in the low temperature section is similar for both formulations, with two current peaks at −100–0°C. According to the above analysis, it is known that −50–0°C is the current peak caused by the interfacial polarization process, and the trap parameters of interfacial polarization are shown in Table 1.

**TABLE 1:** Trap characteristics of interfacial polarization under different formulations.

Sample	Peak Temperature (°C)	Peak current (pA)	Trap depth (eV)	Trapped depth (nC)
EP1/5%HTBN	−22	8.93	0.512	0.305
EP1/15%HTBN	−20	34.31	0.705	0.633
EP1/25%HTBN	−14	67.75	0.841	1.354
EP2/5%HTBN	−22.5	19.39	0.622	0.506
EP2/15%HTBN	−23.5	54.18	0.805	1.202
EP2/25%HTBN	−25	94.85	0.952	1.621

As can be seen from the table, the peak temperature of interfacial polarization peaks with different rubber contents varied less. With the increase of rubber content, the peak current and trap charge of interfacial polarization of EP1 and EP2 increased. The trap energy level and trap charge introduced by the interfacial polarization of EP2 formulation was higher than those of EP1 formulation at the same rubber concentration. This is since HTBN is hardly involved in the curing reaction of the amine formulation system, resulting in a larger amount of rubber precipitation in the EP2 formulation than in the EP1 formulation. Therefore, the rubber filler introduced more traps for the same liquid rubber addition. In addition, due to the difference in epoxy values, the bonding of the epoxy matrix to the rubber particles is also different between the two formulations, resulting in different trap energy levels introduced at the interface. The above differences were also verified in the dielectric spectra (Chen et al., 2020).

### Effect of Hydroxy Liquid Nitrile Rubber Introduction on Electrode Polarization and Conductivity at High Temperature of Epoxy Resin

**FIGURE 8:**
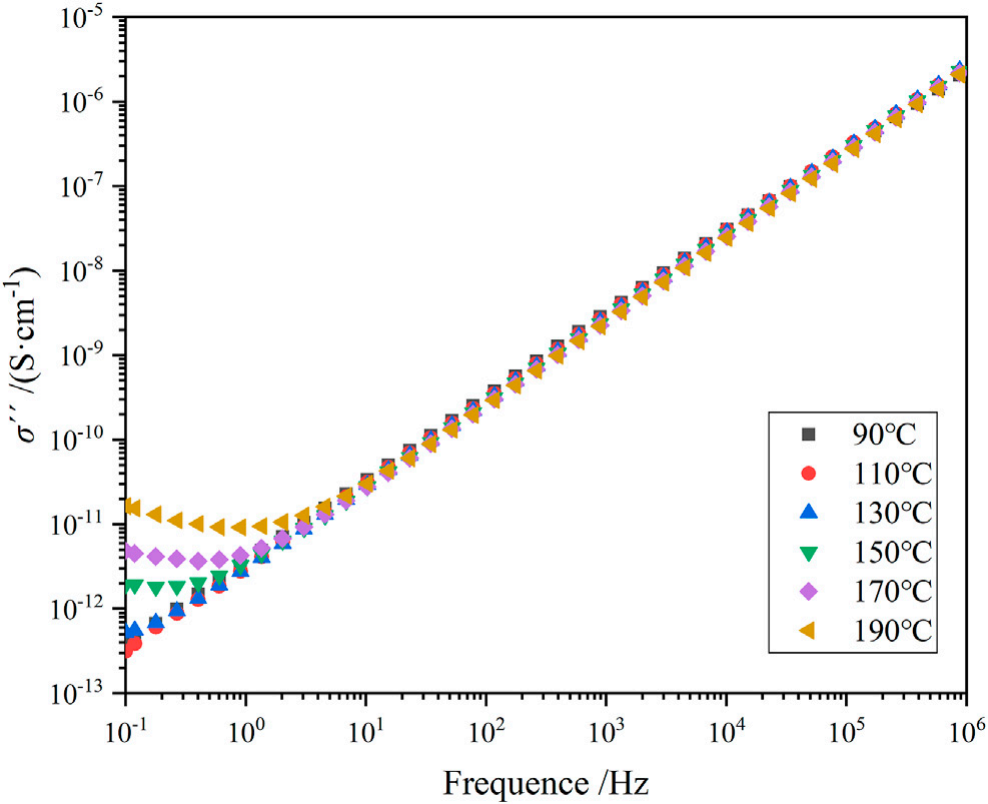
Variation of imaginary part of complex AC conductivity with frequency for pure epoxy samples at different temperatures.

**FIGURE 9:**
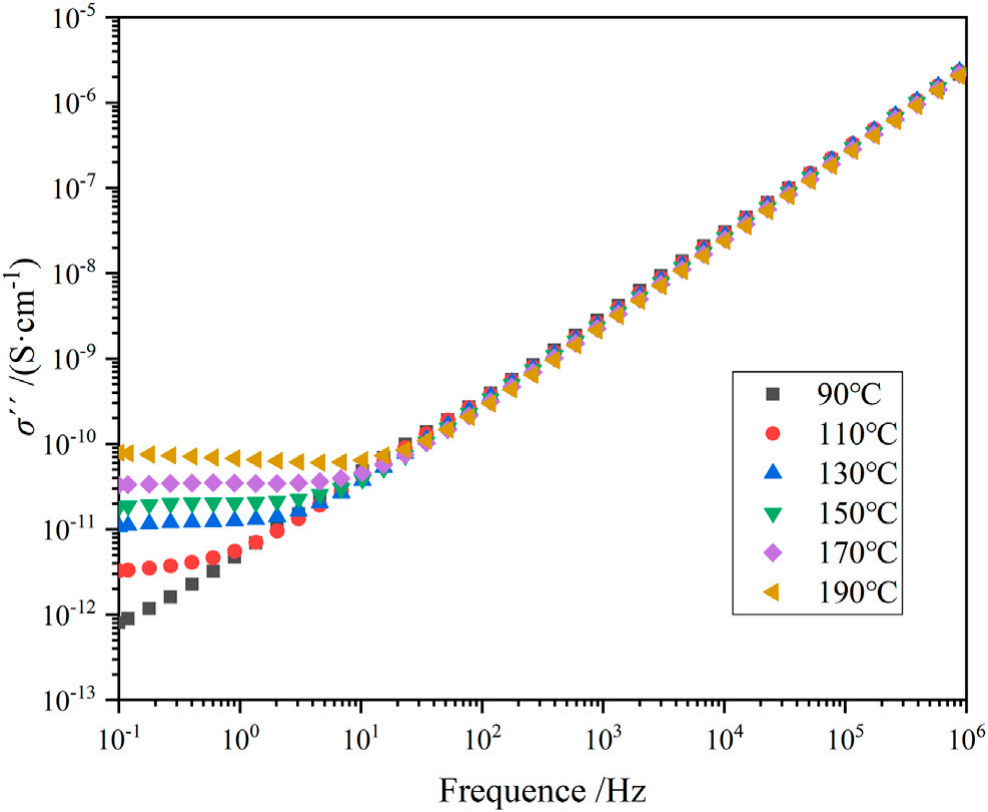
Variation of imaginary part of complex AC conductivity with frequency for 5% HTBN samples at different temperatures.

The glass transition temperature (*T*_g_) of the formulation used in this paper is about 70°C according to the report in literature (Wang et al., 2020b). With the increase of rubber content, the *T*_g_ of the material decreases. According to Figure 3, the TSDC spectrum shows a distinct current peak at around 70°C, which reflects the glass transition process formed by the movement of the epoxy resin molecular chain segments, also called α-relaxation. Figures 8, 9 shows the imaginary part of the complex conductivity variation with frequency for pure epoxy and 25% HTBN specimens at different temperatures respectively. Since the DC conductivity effect only responds at temperatures higher than the glass transition temperature of the specimen. Therefore, only the measurements at temperatures higher than *T*_g_ of the specimens are given in the figure.

As shown in Figures 8, 9, in the logarithmic coordinates, σ′′ increases linearly with increasing frequency for both specimens in the middle and high-frequency regions. The change is little affected by temperature. In the low-frequency region, pure epoxy specimens at a temperature higher than 130°C, σ′′ with frequency decreases the more obvious upward warping. In contrast, rubber-containing specimens at a temperature higher than 90°C appear similar. The reason for the “warping” phenomenon is the beginning of electrode polarization in the specimen (Kremer and Schönhals, 2002). When the pure epoxy specimen temperature is not much higher than the glass transition temperature, the macromolecular chain in the epoxy matrix is not completely thawed. Because the ion transport is assisted by the local motion of the polymer chain segments, the molecular chain segments become active at temperatures higher than *T*_g_. The higher the temperature, the more ions are activated, and many ions accumulate near the electrode. Moreover, the increase of rubber filler introduces a large number of ion carriers, and a more significant concentration of ions accumulates at the electrode. Compared to the pure epoxy specimens, the specimens containing rubber started to show electrode polarization at a lower temperature.

The relationship between complex conductivity and complex permittivity can be obtained according to Maxwell’s equation, the expression of which is shown in Equation 9 (Kremer and Schönhals, 2002).

$$\sigma_{AC}^{*}=\sigma \prime +\text{j}\sigma \prime \prime =2\pi f\varepsilon_{0}\varepsilon^{\prime \prime }+\text{j}2\pi f\varepsilon_{0}\varepsilon^{\prime }$$ (9)

where: *σ*′, *σ*′′ are the real and imaginary components of the complex AC conductivity; *ε*′, *ε*′′ are the real and imaginary parts of the complex permittivity; *ε*_0_ is the vacuum permittivity; *f* is the AC perturbation small signal frequency.

The real part *σ*′ of the AC conductivity and the DC conductivity *σ*_dc_ are in accordance with the Almond-West relationship shown in Equation 10, and the Levenberg-Marquardt algorithm is used to fit the experimental data of the specimens, and the fitting results are shown in Table 2.

$$\sigma^{\prime }\left(f\right)=\sigma_{\text{dc}}\left\{1+{\left(\frac{f}{f_{c}}\right)}^{s}\right\}$$ (10)

Where: *f*_v_ is the test voltage frequency; *f*_c_ is the characteristic frequency, which indicates the shift of the carrier from the motion through the electrode to the reciprocal motion inside the specimen under the action of voltage; *s* is the power index characterizing the material relaxation phenomenon, usually 0 ≤ *s* ≤ 1.

**TABLE 2:** DC conductivity of different samples at high temperature.

Temperature	*σ*_dc_/(S·cm^−1^)			
/°C	NEAT	5%HTBN	15%HTBN	25%HTBN
150	8.584 × 10^−10^	8.616 × 10^−10^	8.579 × 10^−10^	8.348 × 10^−10^
160	1.305 × 10^−10^	1.428 × 10^−10^	1.253 × 10^−9^	1.155 × 10^−9^
170	1.758 × 10^−9^	1.971 × 10^−9^	1.709 × 10^−9^	1.614 × 10^−9^
180	2.952 × 10^−9^	3.337 × 10^−9^	2.648 × 10^−9^	2.337 × 10^−9^
190	3.973 × 10^−9^	4.39 × 10^−9^	3.792 × 10^−9^	3.397 × 10^−9^
200	5.807 × 10^−9^	7.006 × 10^−9^	5.172 × 10^−9^	4.689 × 10^−9^

To further investigate the effect of HTBN on DC conductivity, an Arrhenius fit of DC conductivity was performed using Equation 11, and the fitted parameters were obtained as shown in Table 3.

$$\tau \left(T\right)=A\cdot \exp\left(\frac{-E_{a}}{RT}\right)$$ (11)

where: A is the constant; *E*_a_ is the activation energy; *R* is the gas constant.

**TABLE 3:** Apparent activation energy of the DC conductivity.

HTBN contents/%	*E*_a_/kJ·mol^−1^	*R* ^2^
0	63.851	0.996
5	63.424	0.994
15	60.611	0.996
25	58.115	0.996

As can be seen from Table 3, the activation energy of the DC conductivity process increases and then decreases with the increase of rubber concentration. It is well known that the factors determining the conductivity are the concentration of carriers and the magnitude of mobility. The introduction of HTBN increases the number of carriers in the epoxy matrix on the one hand. On the other hand, the two-phase structure formed will form charge accumulation at the interface under the action of the applied electric field, producing local electric field distortion and playing an influence on the carrier transfer. In combination with Figure 2, the latter dominates when the temperature is higher than 150°C. The size of the interface formed by rubber and epoxy is proportional to the added rubber content, and its hindrance to carrier migration increases. Thus, the σ_dc_ of 5% content specimens reaches the maximum. 150°C, the conductivity of each specimen is close, which is due to the gradual increase of the effect of carrier introduction by HTBN and the gradual decrease of the interfacial effect.

**FIGURE 10:**
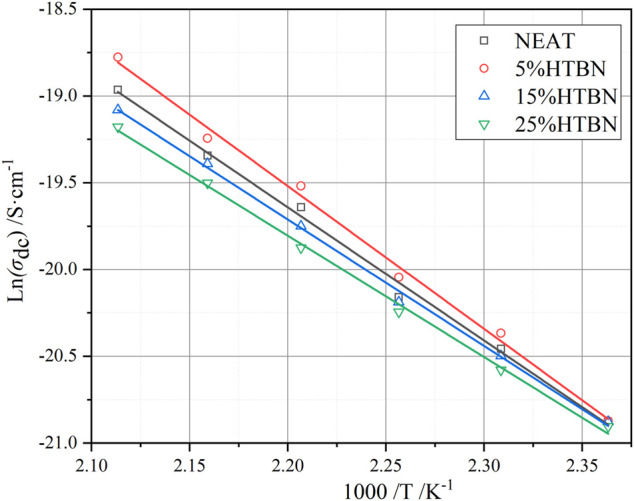
Variation of DC conductivity with temperature for different samples.

From Figure 10, it can be seen that the DC conductivity is by the Arrhenius law, so it can be assumed that the carrier conduction is caused by jumping conductivity. The equivalent decoupling energy level can be expressed as (Ma Chao et al., 2017)

$$\Phi_{\text{h}}=\phi_{\text{h}}-\frac{\text{e}E_{\text{ext}}\lambda }{2}$$ (12)

Where: Φ_h_ is the equivalent delocalization energy level of the hopping conductivity carrier; *φ*_h_ is the trap energy level of the carrier; *E*_ext_ is the field strength of the externally applied electric field, and λ is the average free range of the electron.

**FIGURE 11:**
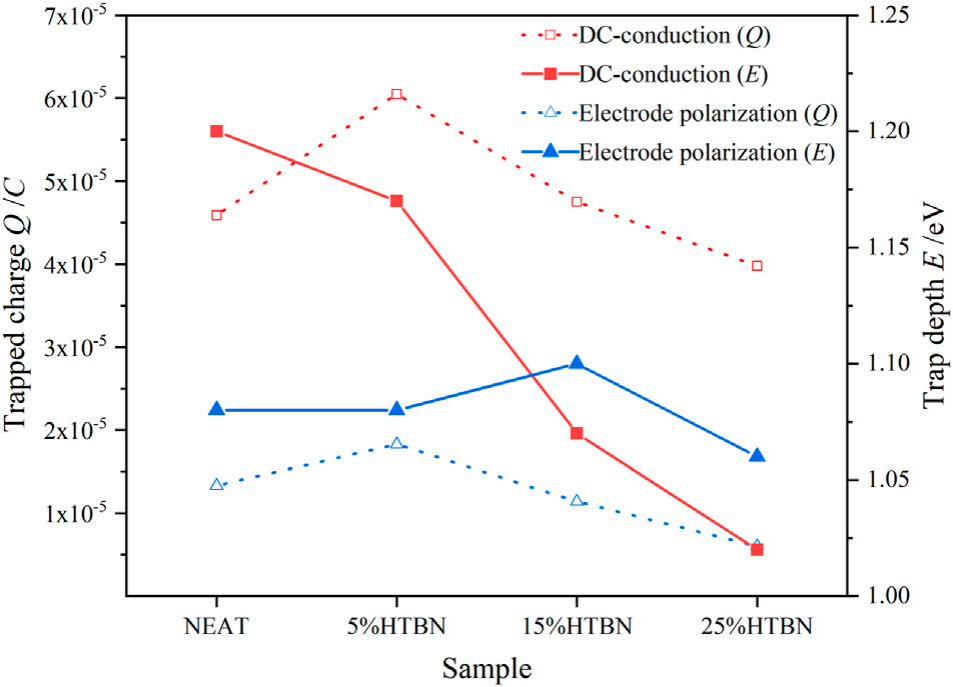
Trap characteristics of DC conductance and electrode polarization of samples with different rubber content.

According to Equation 12, the trap energy level is positively correlated with the carrier equivalent delocalization energy level. Therefore, when the trap energy level decreases, the trap trapping effect is weakened. According to the bipolar charge transport model (Xie et al., 2018), when the trap trapping effect is weakened, the average free range of the carriers increases, leading to higher mobility and energy. As shown in Figure 11, the trap energy level of the DC conductor decreases with the increase of the rubber content. The larger the trapped energy level, the energy gained from the electric field as the carriers migrate in the free volume does not readily cross the deep trap potential barrier, thus reducing the number density and mobility of migrating carriers. However, trap energy level and trapped charge of electrode polarization vary less with increasing rubber content.

## Conclusion

In this work, TSDC and broadband dielectric spectroscopy techniques were used to complementarily characterize the dielectric relaxation processes of end-hydroxy liquid nitrile rubber (HTBN) toughened epoxy resin polymers and the trap characteristics of each polarization process were analyzed, leading to the following conclusions:

HTBN introduces two new relaxation processes in the composite. Rubber molecular α polarization and interfacial polarization, both trap energy levels, are proportional to the rubber concentration. The interfacial polarization introduces trap energy levels from 0.5 to 0.9 eV. The introduction of HTBN has a negligible effect on the trap characteristics of epoxy resin’s high-temperature electrode polarization process due to the conductivity effect. The trapped charge of electrode polarization is maximum at 5% HTBN content. DC conductivity is the main factor leading to the variation of the dielectric loss factor in the high-temperature region. The variation with temperature follows the Arrhenius law, whose activation energy increases first and then decreases with increasing rubber concentration and whose trap energy level decreases with increasing rubber concentration.

## Data Availability

The original contributions presented in the study are included in the article/Supplementary Material, further inquiries can be directed to the corresponding author.
